# 4-Hy­droxy-*N*′-(3-nitro­benzyl­idene)benzohydrazide

**DOI:** 10.1107/S1600536812014778

**Published:** 2012-04-13

**Authors:** Jin-Long Hou

**Affiliations:** a College of Chemistry and Chemical Engineering, Qiqihar University, Qiqihar 161006, People’s Republic of China

## Abstract

The title compound, C_14_H_11_N_3_O_4_, was obtained by a condensation reaction between 3-nitro­benzaldehyde and 4-hy­droxy­benzohydrazide. The whole mol­ecule is approximately planar, with a dihedral angle of 9.2 (3)° between the benzene rings. The mol­ecule displays an *E* conformation about the C=N bond. In the crystal, mol­ecules are linked *via* N—H⋯O, O—H⋯O and O—H⋯N hydrogen bonds, generating sheets parallel to the *bc* plane.

## Related literature

For the biological properties of hydrazone compounds, see: Cukurovali *et al.* (2006 ▶); Karthikeyan *et al.* (2006 ▶); Kucukguzel *et al.* (2006 ▶). For related hydrazone compounds, see: Hou (2009 ▶); Mohd Lair *et al.* (2009 ▶); Fun *et al.* (2008 ▶); Zhang *et al.* (2009 ▶); Khaledi *et al.* (2008 ▶). For standard bond lengths, see: Allen *et al.* (1987) ▶.
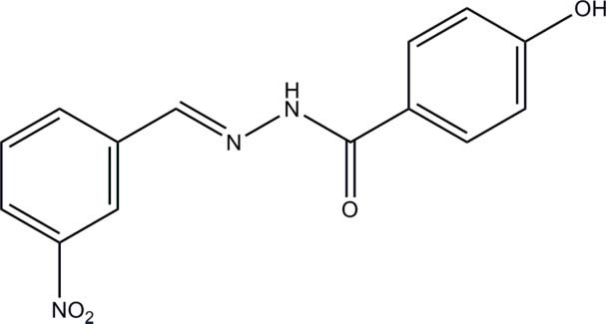


## Experimental

### 

#### Crystal data

C_14_H_11_N_3_O_4_
*M* *_r_* = 285.26
Monoclinic, 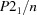

*a* = 8.018 (2) Å
*b* = 11.156 (2) Å
*c* = 14.389 (2) Å
β = 91.773 (2)°
*V* = 1286.4 (5) Å^3^
*Z* = 4
Mo *K*α radiation
μ = 0.11 mm^−1^
*T* = 298 K
0.21 × 0.20 × 0.17 mm

#### Data collection

Bruker SMART 1000 CCD area-detector diffractometer
Absorption correction: multi-scan (*SADABS*; Sheldrick, 1996 ▶) *T*_min_ = 0.977, *T*_max_ = 0.981
9218 measured reflections
2386 independent reflections
2020 reflections with *I* > 2σ(*I*)
*R*_int_ = 0.018

#### Refinement

*R*[*F* ^2^ > 2σ(*F* ^2^)] = 0.035
*wR*(*F* ^2^) = 0.108
*S* = 1.10
2386 reflections
194 parameters
1 restraint
H atoms treated by a mixture of independent and constrained refinement
Δρ_max_ = 0.13 e Å^−3^
Δρ_min_ = −0.23 e Å^−3^

### 

Data collection: *SMART* (Bruker, 1998 ▶); cell refinement: *SAINT* (Bruker, 1998 ▶); data reduction: *SAINT*; program(s) used to solve structure: *SHELXS97* (Sheldrick, 2008 ▶); program(s) used to refine structure: *SHELXL97* (Sheldrick, 2008 ▶); molecular graphics: *SHELXTL* (Sheldrick, 2008 ▶); software used to prepare material for publication: *SHELXTL*.

## Supplementary Material

Crystal structure: contains datablock(s) global, I. DOI: 10.1107/S1600536812014778/qm2061sup1.cif

Structure factors: contains datablock(s) I. DOI: 10.1107/S1600536812014778/qm2061Isup2.hkl

Supplementary material file. DOI: 10.1107/S1600536812014778/qm2061Isup3.cml

Additional supplementary materials:  crystallographic information; 3D view; checkCIF report

## Figures and Tables

**Table 1:** Hydrogen-bond geometry (Å, °)

*D*—H⋯*A*	*D*—H	H⋯*A*	*D*⋯*A*	*D*—H⋯*A*
N2—H2*A*⋯O1^i^	0.90 (1)	2.49 (2)	3.0406 (18)	120 (1)
N2—H2*A*⋯O4^ii^	0.90 (1)	2.32 (1)	3.0360 (17)	137 (2)
O4—H4⋯N1^iii^	0.82	2.63	3.0495 (17)	114
O4—H4⋯O3^iii^	0.82	2.08	2.8929 (16)	173

Symmetry codes: (i) 
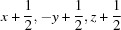
; (ii) 
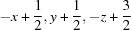
; (iii) 
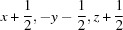
.

**Fig. 1.**
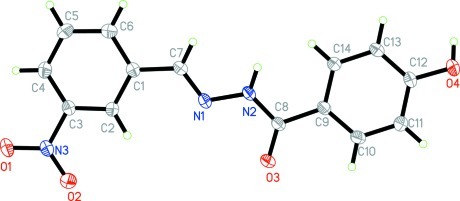
Molecular structure with displacement ellipsoids drawn at 30% probability for non-H atoms.

**Fig. 2.**
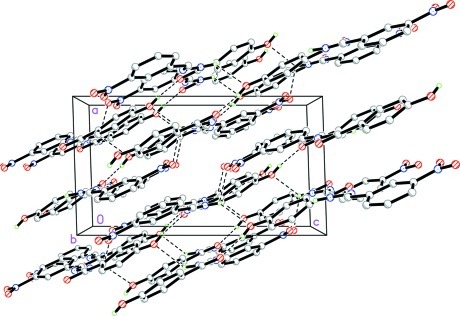
Molecular packing diagram, viewed along the b axis. Hydrogen bonds are shown as dashed lines.

## supplementary crystallographic information

### Comment

Hydrazones derived from the condensation reactions of hydrazides with aldehydes show excellent biological properties (Cukurovali *et al.*, 2006; Karthikeyan *et al.*, 2006; Kucukguzel *et al.*, 2006). In the last few years, a great deal of hydrazone compounds have been reported for their crystal structures see (Hou, 2009; Mohd Lair *et al.*, 2009; Fun *et al.*, 2008; Zhang *et al.*, 2009; Khaledi *et al.*, 2008). In this paper, the title new compound, derived from the condensation reaction of 3-nitrobenzaldehyde and 4-hydroxybenzohydrazide was synthesized and structurally characterized.

The molecular structure of the compound is shown in Fig. 1. The whole molecule of the compound is approximately coplanar, with the dihedral angle between the mean planes through the two benzene rings of 9.2 (3)°. The molecule displays an *E* configuration about the C=N bond. All the bond lengths are within normal ranges (Allen *et al.*, 1987). In the crystal, molecules are linked *via* N–H···O hydrogen bonds (Table 1), generating two-dimensional sheets (Fig. 2).

### Experimental

3-Nitrobenzaldehyde (1.0 mmol, 151 mg) and 4-hydroxybenzohydrazide (1.0 mmol, 152 mg) were mixed and refluxed with stirring for two hours. Yellow single crystals were formed after slow evaporation of the solution in air for a week.

### Refinement

H2A was located in a difference Fourier map and refined isotropically, with the N–H distance restrained to 0.90 (1) Å. The other H atoms were placed in idealized positions and constrained to ride on their parent atoms with C–H distances of 0.93 Å, O–H distance of 0.82 Å, and with *U*_iso_(H) set at 1.2*U*_eq_(C) and 1.5*U*_eq_(O).

### Crystal data

**Table:**

C_14_H_11_N_3_O_4_	*F*(000) = 592
*M**_r_* = 285.26	*D*_x_ = 1.473 Mg m^−^^3^
Monoclinic, *P*2_1_/*n*	Mo *K*α radiation, λ = 0.71073 Å
*a* = 8.018 (2) Å	Cell parameters from 4759 reflections
*b* = 11.156 (2) Å	θ = 2.8–27.0°
*c* = 14.389 (2) Å	µ = 0.11 mm^−^^1^
β = 91.773 (2)°	*T* = 298 K
*V* = 1286.4 (5) Å^3^	Block, yellow
*Z* = 4	0.21 × 0.20 × 0.17 mm

### Data collection

**Table:**

Bruker SMART 1000 CCD area-detector diffractometer	2386 independent reflections
Radiation source: fine-focus sealed tube	2020 reflections with *I* > 2σ(*I*)
Graphite monochromator	*R*_int_ = 0.018
ω scans	θ_max_ = 25.5°, θ_min_ = 2.9°
Absorption correction: multi-scan (*SADABS*; Sheldrick, 1996)	*h* = −9→9
*T*_min_ = 0.977, *T*_max_ = 0.981	*k* = −13→13
9218 measured reflections	*l* = −17→17

### Refinement

**Table:**

Refinement on *F*^2^	Primary atom site location: structure-invariant direct methods
Least-squares matrix: full	Secondary atom site location: difference Fourier map
*R*[*F*^2^ > 2σ(*F*^2^)] = 0.035	Hydrogen site location: inferred from neighbouring sites
*wR*(*F*^2^) = 0.108	H atoms treated by a mixture of independent and constrained refinement
*S* = 1.10	*w* = 1/[σ^2^(*F*_o_^2^) + (0.0549*P*)^2^ + 0.347*P*] where *P* = (*F*_o_^2^ + 2*F*_c_^2^)/3
2386 reflections	(Δ/σ)_max_ < 0.001
194 parameters	Δρ_max_ = 0.13 e Å^−^^3^
1 restraint	Δρ_min_ = −0.23 e Å^−^^3^

### Special details

**Table:**

Geometry. All e.s.d.'s (except the e.s.d. in the dihedral angle between two l.s. planes) are estimated using the full covariance matrix. The cell e.s.d.'s are taken into account individually in the estimation of e.s.d.'s in distances, angles and torsion angles; correlations between e.s.d.'s in cell parameters are only used when they are defined by crystal symmetry. An approximate (isotropic) treatment of cell e.s.d.'s is used for estimating e.s.d.'s involving l.s. planes.
Refinement. Refinement of *F*^2^ against ALL reflections. The weighted *R*-factor *wR* and goodness of fit *S* are based on *F*^2^, conventional *R*-factors *R* are based on *F*, with *F* set to zero for negative *F*^2^. The threshold expression of *F*^2^ > σ(*F*^2^) is used only for calculating *R*-factors(gt) *etc*. and is not relevant to the choice of reflections for refinement. *R*-factors based on *F*^2^ are statistically about twice as large as those based on *F*, and *R*- factors based on ALL data will be even larger.

### Fractional atomic coordinates and isotropic or equivalent isotropic displacement parameters (Å^2^)

**Table:**

	*x*	*y*	*z*	*U*_iso_*/*U*_eq_	
N1	0.18378 (15)	0.04393 (11)	0.44521 (8)	0.0361 (3)	
N2	0.21274 (16)	−0.03537 (12)	0.51683 (8)	0.0372 (3)	
N3	0.02101 (16)	0.30324 (12)	0.15979 (9)	0.0405 (3)	
O1	−0.00751 (15)	0.39126 (11)	0.11049 (8)	0.0533 (3)	
O2	−0.01799 (18)	0.20157 (11)	0.13697 (9)	0.0610 (4)	
O3	0.14307 (14)	−0.19871 (10)	0.43108 (7)	0.0420 (3)	
O4	0.43051 (15)	−0.43925 (11)	0.80509 (8)	0.0477 (3)	
H4	0.4981	−0.4032	0.8386	0.072*	
C1	0.18748 (18)	0.24891 (14)	0.39868 (10)	0.0357 (3)	
C2	0.11420 (18)	0.22848 (13)	0.31117 (10)	0.0348 (3)	
H2	0.0733	0.1532	0.2946	0.042*	
C3	0.10429 (18)	0.32288 (13)	0.25017 (10)	0.0349 (3)	
C4	0.1649 (2)	0.43600 (15)	0.27064 (12)	0.0435 (4)	
H4A	0.1576	0.4974	0.2270	0.052*	
C5	0.2364 (2)	0.45524 (15)	0.35766 (13)	0.0499 (4)	
H5	0.2780	0.5306	0.3735	0.060*	
C6	0.2463 (2)	0.36266 (15)	0.42129 (12)	0.0453 (4)	
H6	0.2930	0.3767	0.4802	0.054*	
C7	0.20456 (19)	0.15322 (14)	0.46738 (10)	0.0379 (4)	
H7	0.2313	0.1727	0.5289	0.045*	
C8	0.20226 (17)	−0.15485 (14)	0.50337 (9)	0.0329 (3)	
C9	0.26711 (17)	−0.22804 (13)	0.58334 (9)	0.0331 (3)	
C10	0.22900 (19)	−0.34905 (14)	0.58765 (10)	0.0389 (4)	
H10	0.1649	−0.3839	0.5399	0.047*	
C11	0.2843 (2)	−0.41868 (14)	0.66141 (11)	0.0415 (4)	
H11	0.2574	−0.4997	0.6633	0.050*	
C12	0.38050 (18)	−0.36750 (14)	0.73294 (10)	0.0358 (3)	
C13	0.4214 (2)	−0.24795 (15)	0.72935 (11)	0.0447 (4)	
H13	0.4862	−0.2135	0.7770	0.054*	
C14	0.3661 (2)	−0.17939 (15)	0.65496 (11)	0.0446 (4)	
H14	0.3956	−0.0989	0.6526	0.054*	
H2A	0.231 (2)	−0.0045 (16)	0.5739 (8)	0.054*	

### Atomic displacement parameters (Å^2^)

**Table:**

	*U*^11^	*U*^22^	*U*^33^	*U*^12^	*U*^13^	*U*^23^
N1	0.0422 (7)	0.0365 (7)	0.0290 (6)	0.0028 (5)	−0.0068 (5)	0.0051 (5)
N2	0.0512 (7)	0.0345 (7)	0.0253 (6)	0.0020 (6)	−0.0102 (5)	0.0017 (5)
N3	0.0406 (7)	0.0410 (8)	0.0394 (7)	0.0042 (6)	−0.0070 (5)	0.0063 (6)
O1	0.0567 (7)	0.0501 (7)	0.0519 (7)	0.0071 (6)	−0.0149 (6)	0.0157 (6)
O2	0.0864 (10)	0.0441 (8)	0.0509 (7)	−0.0059 (7)	−0.0252 (7)	−0.0004 (6)
O3	0.0542 (7)	0.0405 (6)	0.0304 (5)	−0.0024 (5)	−0.0128 (5)	−0.0017 (5)
O4	0.0570 (7)	0.0467 (7)	0.0386 (6)	−0.0020 (5)	−0.0126 (5)	0.0146 (5)
C1	0.0363 (7)	0.0347 (8)	0.0357 (8)	0.0015 (6)	−0.0037 (6)	0.0021 (6)
C2	0.0366 (7)	0.0302 (8)	0.0372 (8)	0.0014 (6)	−0.0036 (6)	0.0014 (6)
C3	0.0345 (7)	0.0347 (8)	0.0353 (8)	0.0033 (6)	−0.0031 (6)	0.0026 (6)
C4	0.0497 (9)	0.0354 (9)	0.0453 (9)	−0.0014 (7)	−0.0030 (7)	0.0089 (7)
C5	0.0612 (10)	0.0340 (9)	0.0541 (10)	−0.0117 (8)	−0.0063 (8)	0.0002 (8)
C6	0.0522 (9)	0.0416 (9)	0.0415 (9)	−0.0062 (7)	−0.0093 (7)	−0.0016 (7)
C7	0.0423 (8)	0.0382 (9)	0.0324 (8)	−0.0004 (6)	−0.0091 (6)	0.0006 (6)
C8	0.0339 (7)	0.0373 (8)	0.0273 (7)	0.0000 (6)	−0.0033 (5)	0.0007 (6)
C9	0.0352 (7)	0.0359 (8)	0.0278 (7)	0.0013 (6)	−0.0038 (6)	0.0013 (6)
C10	0.0455 (8)	0.0364 (9)	0.0340 (8)	−0.0002 (6)	−0.0085 (6)	−0.0039 (6)
C11	0.0511 (9)	0.0313 (8)	0.0415 (9)	−0.0017 (7)	−0.0064 (7)	0.0029 (7)
C12	0.0374 (7)	0.0392 (9)	0.0305 (7)	0.0042 (6)	−0.0019 (6)	0.0068 (6)
C13	0.0524 (9)	0.0422 (9)	0.0382 (8)	−0.0051 (7)	−0.0178 (7)	0.0033 (7)
C14	0.0545 (9)	0.0357 (9)	0.0424 (9)	−0.0079 (7)	−0.0183 (7)	0.0062 (7)

### Geometric parameters (Å, º)

**Table:**

N1—C7	1.270 (2)	C4—H4A	0.9300
N1—N2	1.3725 (17)	C5—C6	1.381 (2)
N2—C8	1.349 (2)	C5—H5	0.9300
N2—H2A	0.898 (9)	C6—H6	0.9300
N3—O2	1.2188 (18)	C7—H7	0.9300
N3—O1	1.2286 (17)	C8—C9	1.491 (2)
N3—C3	1.460 (2)	C9—C10	1.386 (2)
O3—C8	1.2312 (17)	C9—C14	1.392 (2)
O4—C12	1.3614 (17)	C10—C11	1.377 (2)
O4—H4	0.8200	C10—H10	0.9300
C1—C6	1.389 (2)	C11—C12	1.390 (2)
C1—C2	1.392 (2)	C11—H11	0.9300
C1—C7	1.458 (2)	C12—C13	1.375 (2)
C2—C3	1.372 (2)	C13—C14	1.378 (2)
C2—H2	0.9300	C13—H13	0.9300
C3—C4	1.381 (2)	C14—H14	0.9300
C4—C5	1.378 (2)		
			
C7—N1—N2	114.35 (12)	C1—C6—H6	119.5
C8—N2—N1	121.38 (12)	N1—C7—C1	121.57 (14)
C8—N2—H2A	121.2 (12)	N1—C7—H7	119.2
N1—N2—H2A	117.3 (13)	C1—C7—H7	119.2
O2—N3—O1	123.13 (14)	O3—C8—N2	122.31 (13)
O2—N3—C3	119.08 (13)	O3—C8—C9	123.39 (14)
O1—N3—C3	117.79 (13)	N2—C8—C9	114.30 (12)
C12—O4—H4	109.5	C10—C9—C14	117.92 (14)
C6—C1—C2	119.49 (14)	C10—C9—C8	119.72 (13)
C6—C1—C7	119.08 (14)	C14—C9—C8	122.36 (14)
C2—C1—C7	121.44 (14)	C11—C10—C9	121.17 (14)
C3—C2—C1	117.89 (14)	C11—C10—H10	119.4
C3—C2—H2	121.1	C9—C10—H10	119.4
C1—C2—H2	121.1	C10—C11—C12	119.81 (15)
C2—C3—C4	123.52 (14)	C10—C11—H11	120.1
C2—C3—N3	118.08 (14)	C12—C11—H11	120.1
C4—C3—N3	118.38 (13)	O4—C12—C13	122.28 (14)
C5—C4—C3	118.04 (15)	O4—C12—C11	117.83 (14)
C5—C4—H4A	121.0	C13—C12—C11	119.89 (14)
C3—C4—H4A	121.0	C12—C13—C14	119.76 (15)
C4—C5—C6	119.98 (16)	C12—C13—H13	120.1
C4—C5—H5	120.0	C14—C13—H13	120.1
C6—C5—H5	120.0	C13—C14—C9	121.43 (15)
C5—C6—C1	121.07 (15)	C13—C14—H14	119.3
C5—C6—H6	119.5	C9—C14—H14	119.3

### Hydrogen-bond geometry (Å, º)

**Table:**

*D*—H···*A*	*D*—H	H···*A*	*D*···*A*	*D*—H···*A*
N2—H2*A*···O1^i^	0.90 (1)	2.49 (2)	3.0406 (18)	120 (1)
N2—H2*A*···O4^ii^	0.90 (1)	2.32 (1)	3.0360 (17)	137 (2)
O4—H4···N1^iii^	0.82	2.63	3.0495 (17)	114
O4—H4···O3^iii^	0.82	2.08	2.8929 (16)	173

Symmetry codes: (i) *x*+1/2, −*y*+1/2, *z*+1/2; (ii) −*x*+1/2, *y*+1/2, −*z*+3/2; (iii) *x*+1/2, −*y*−1/2, *z*+1/2.
